# Inorganic Tricarbonate: High-Pressure Synthesis and Structure of K_2_C_3_O_7_

**DOI:** 10.1021/jacs.5c19614

**Published:** 2026-03-24

**Authors:** Andrey Aslandukov, Alena Aslandukova, Fariia I. Akbar, Yuqing Yin, Ridvan Akilli, Gaston Garbarino, Dominik Spahr, Bjoern Winkler, Maxim Bykov, Natalia Dubrovinskaia, Leonid Dubrovinsky

**Affiliations:** † Institute of Inorganic and Analytical Chemistry, 9173 Goethe University Frankfurt, Frankfurt 60438, Germany; ‡ Material Physics and Technology at Extreme Conditions, Laboratory of Crystallography, 26523 University of Bayreuth, Bayreuth 95440, Germany; § European Synchrotron Radiation Facility, Grenoble 38000, France; ∥ Institute of Geosciences, Goethe University Frankfurt, Frankfurt 60438, Germany; ⊥ Bavarian Research Institute of Experimental Geochemistry and Geophysics (BGI), University of Bayreuth, Bayreuth 95440, Germany

## Abstract

The synthesis of carbonates with novel types of anions is important for geoscience, chemistry, and materials science. Herein, we present the first inorganic tricarbonate salt, K_2_C_3_O_7_, discovered in laser-heated diamond anvil cells at 55(3) and 45(2) GPa. The crystal structure of K_2_C_3_O_7_ was determined in situ by using synchrotron single-crystal X-ray diffraction from a polycrystalline sample. It features nonplanar [C_3_O_7_]^2–^ anions, which consist of three corner-sharing planar CO_3_ groups rotated relative to one another. This anion extends the homologous series of sp^2^-carbonates: [CO_3_]^2–^—[C_2_O_5_]^2–^—[C_3_O_7_]^2–^. Raman spectroscopy establishes the characteristic vibrational fingerprint of the [C_3_O_7_]^2–^ anion. Density functional theory (DFT) calculations corroborate the experimental results and suggest a thermodynamic stability of K_2_C_3_O_7_ between 10 and 55 GPa. DFT calculations predict a phase transition between 80 and 90 GPa associated with polymerization of the [C_3_O_7_]^2–^ groups, accompanied by a change in the coordination polyhedra of two carbon atoms from triangles to tetrahedra. These results imply that other sp^2^-and mixed sp^2^/sp^3^-carbonates might be stabilized at a high pressure.

## Graphical abstract

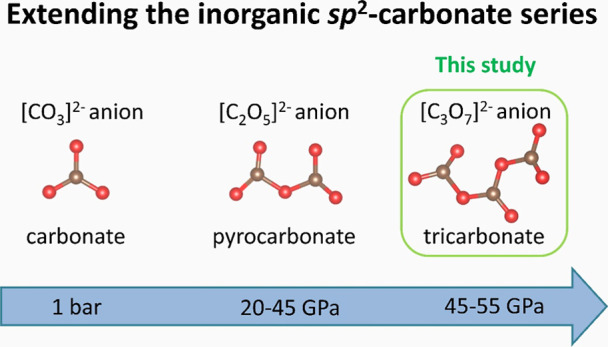

## Introduction

Inorganic carbonates represent an important class of materials, studied for fundamental and applied science. At ambient conditions, carbonates feature planar trigonal [CO_3_]^2–^ groups. High pressure promotes the stabilization of more complex carbonate anions. Among them are sp^3^-carbonate anions built of CO_4_ tetrahedra—isolated^1–3^ or connected to other tetrahedra by sharing corners, forming [C_3_O_9_]^6–^ rings,^4–6^ four-membered [C_4_O_10_]^4–^ pyramidal units,^7–9^ four-membered [C_4_O_13_]^10–^ chains,^1,10^ or infinite chains.^11^ Besides the sp^3^-carbonates built of CO_4_ units, sp^2^-carbonates represented by pyrocarbonates featuring [C_2_O_5_]^2–^ anions have also been discovered under high pressure. After the first stabilization of the [C_2_O_5_]^2–^ anion in SrC_2_O_5_ at 20 GPa reported in 2022,^12^ the family of pyrocarbonates grew, and pyrocarbonates with mono-, di-, and trivalent metal cations have been obtained in the pressure range of 20–46 GPa.^13–17^ These discoveries show that the planar CO_3_ unit, along with the tetrahedral CO_4_ unit, can potentially also be a building block of exotic C–O anions at a high pressure. In this sense, there is a possible parallel with borate chemistry, where numerous complex anions comprised of BO_3_ and BO_4_ building blocks are known.^18^ However, except for the [C_2_O_5_]^2–^ anion, no other anions containing CO_3_ units are known so far.

Here, we report the discovery of K_2_C_3_O_7_ featuring tricarbonate [C_3_O_7_]^2–^ anions comprised of three condensed CO_3_ groups. The compound was synthesized under high-pressure, high-temperature conditions and characterized via synchrotron single-crystal X-ray diffraction, Raman spectroscopy, and density functional theory (DFT).

## Results and Discussion

K_2_C_3_O_7_ was initially observed serendipitously at 55(3) GPa during the exploration of potassium chlorate behavior at high-pressure high-temperature conditions (see Supporting Information discussion 1 for details). Later, the compound was synthesized targetedly from K_2_CO_3_ and CO_2_ at 45(2) GPa and 3000 K. The observation of K_2_C_3_O_7_ in different chemical environments and at different pressures indicates its thermodynamic stability across a broad range of chemical potentials and P–T conditions.

In both cases, the formation of K_2_C_3_O_7_ was identified by synchrotron single-crystal X-ray diffraction data collected from a multigrain sample (Figure 1a). For the direct synthesis at 45(2) GPa, K_2_C_3_O_7_ was additionally characterized by Raman spectroscopy (Supporting Information discussion 2). Refinement against the single-crystal X-ray diffraction data yielded very good agreement factors (Tables S1 and S2). Below, the crystal structure at 55(3) GPa is discussed. The structure of K_2_C_3_O_7_ has a monoclinic space group *P*2_1_/*c* (#14) with the lattice parameters *a* = 4.9424(13) Å, *b* = 10.946(2) Å, *c* = 7.243(2) Å, β = 105.02(3)°, and volume *V* = 378.44(18) Å^3^ at 55(3) GPa. K_2_C_3_O_7_ crystallizes in a novel structure type with two K, three C, and seven O symmetrically independent atoms, all occupying 4e Wyckoff positions (Figure 1b). The structure of K_2_C_3_O_7_ features [C_3_O_7_]^2–^ tricarbonate anions that are aligned in stacks along the *a* direction (Figure 1c). Potassium atoms are located between the stacks. The K1 atom possesses a coordination number of CN = 11 with an average K1–O distance of 2.474(28) Å at 55(3) GPa. The K2 atom exhibits a coordination number of CN = 10 with an average K2–O distance of 2.422(25) Å at 55(3) GPa.

**Figure 1:**
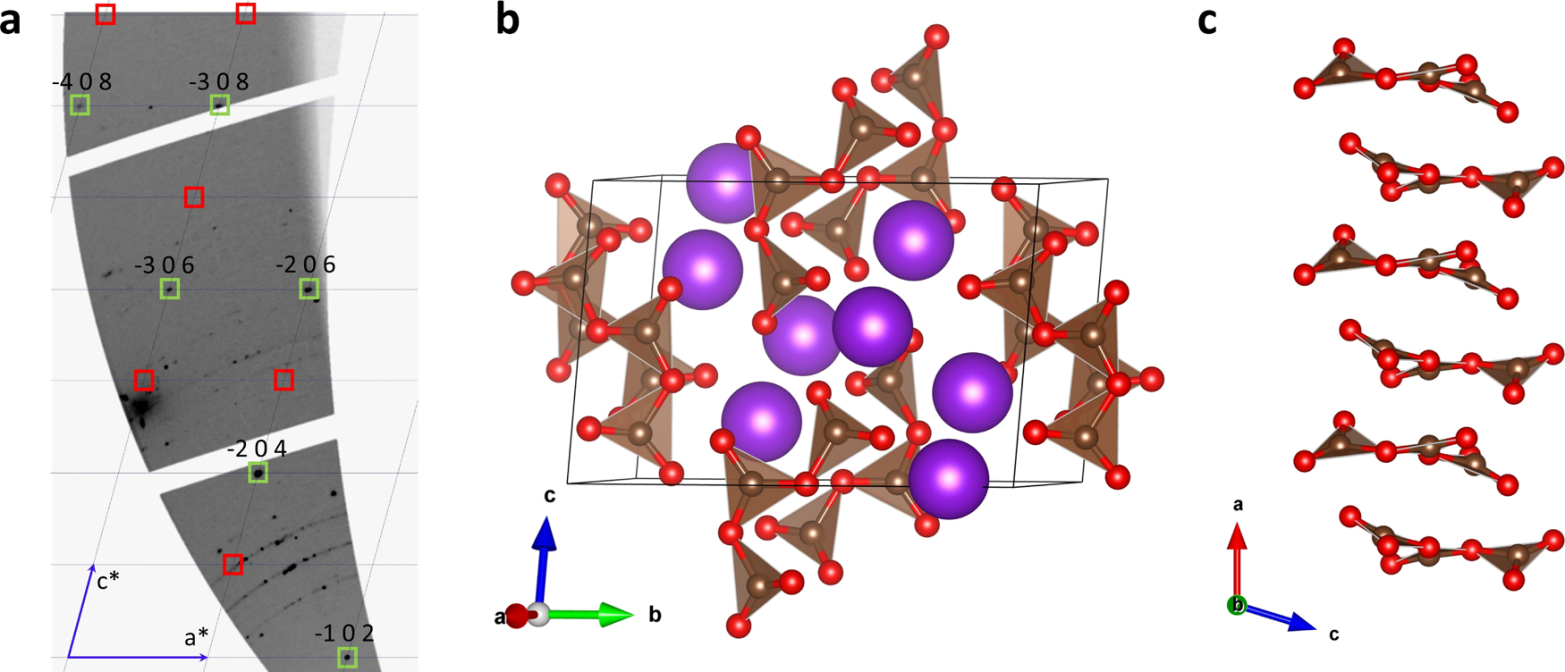
(a) Part of the reconstructed (*h 0 l*) reciprocal lattice plane of K_2_C_3_O_7_ at 55(3) GPa from the experimental single-crystal XRD data set obtained using CrysAlis^Pro^ software. The green-labeled reflections correspond to the crystallite of K_2_C_3_O_7_ whose structure was determined. Red labels correspond to the positions of the *h* 0 *l* reflections with *l* = 2n + 1, which are systematically absent in the *P*2_1_/*c* space group. (b) General view of the crystal structure of K_2_C_3_O_7_ at 55(3) GPa. (c) Stack of [C_3_O_7_]^2–^ groups along the *a* direction.

The [C_3_O_7_]^2–^ anion is nonplanar and can be described as three planar CO_3_ groups connected through bridging oxygen atoms (O3 and O5, Figure 2). The planarity of the CO_3_ units indicates a sp^2^ hybridization of the carbon atoms, although the ∠O–C–O angles within the CO_3_ units deviate from the ideal 120° (Figure 2a). Bridging ∠C1–O3–C2 and ∠C2–O5–C3 angles are almost 120°, suggesting the sp^2^ hybridization of bridging oxygen atoms (Figure 2a). The lengths of the terminal C1–O1, C1–O2, C3–O6, and C3–O7 bonds (ranging from 1.205(7) to 1.218(7) Å) are similar to those observed in pyrocarbonates.^12–17^ In contrast to pyrocarbonates, however, both bridging oxygen atoms O3 and O5 in the [C_3_O_7_]^2–^ anion form one shorter bond with the middle C2 atom (1.283(7) and 1.288(7) Å, respectively) and one significantly longer O3–C1 or O5–C3 bond with terminal carbon atoms (1.450(8) and 1.401(8) Å, respectively), suggesting π-bonding with the C2 atom and σ single bonding with terminal C1 and C3 carbon atoms (Figure 2a). The observed bond length and corresponding bond orders of all C–O bonds within the [C_3_O_7_]^2–^ anion (Table S3) can be rationalized within the framework of classical valence bond theory by considering all possible resonance forms of the [C_3_O_7_]^2–^ anion (Figure S1).

**Figure 2:**
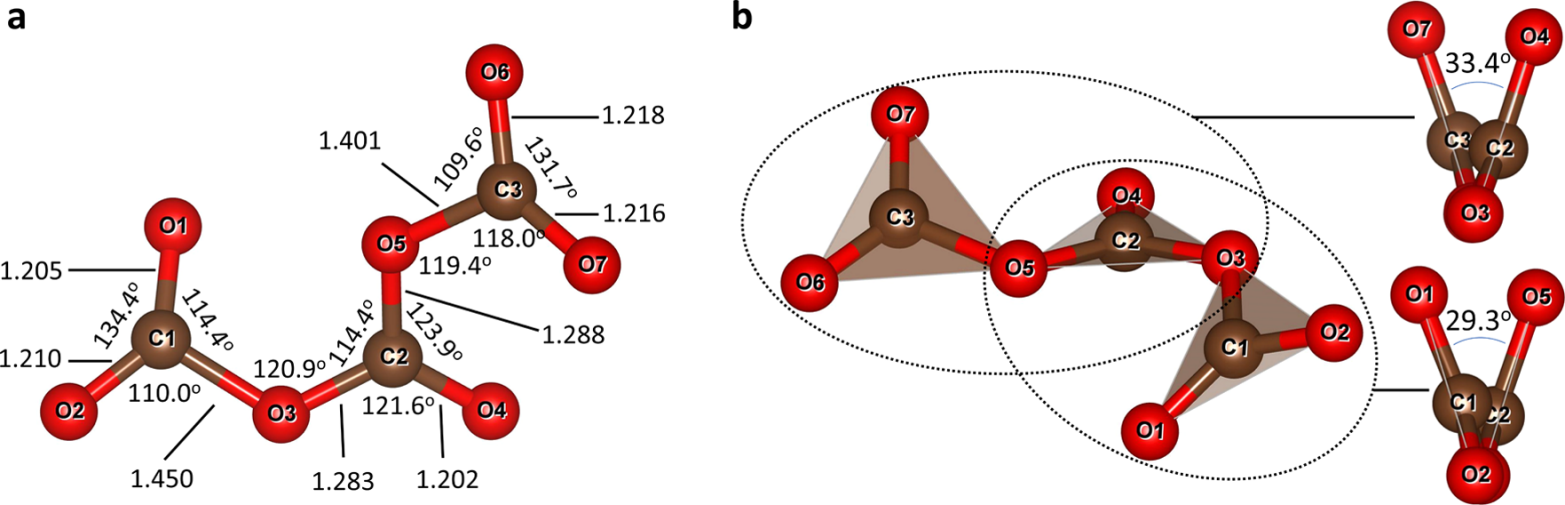
Geometry of the [C_3_O_7_]^2–^ anion. (a) The [C_3_O_7_]^2–^ group with indicated C–O bond distances, ∠C–O–C and ∠O–C–O angles. (b) Tilting of the CO_3_ triangular units in the [C_3_O_7_]^2–^ anion. The dihedral angles represent the tilting between pairs of the respective planar CO_3_ groups, as viewed along the O2–O3–O4 and O3–O5–O6 directions.

In the idealized case, this anion may adopt a planar geometry with a conjugated π-system (Figure S1). In practice, however, it is nonplanar—terminal CO_3_ units are rotated with respect to the middle CO_3_ unit— most likely due to favorable packing under high pressure. Interestingly, the atoms O2, O3, and O4 atoms are nearly collinear (∠O2–O3–O4 = 178°), as are atoms O3, O5, and O6 (∠O3–O5–O6 = 177°), indicating negligibly minimal bending at the bridging oxygen atoms (Figure 2b). Two CO_3_ groups are rotated relative to each other by 29.3° around the O2–O3–O4 axis and another two CO_3_ groups by 33.4° around the O3–O5–O6 axis (Figure 2b).

It is well-established by now that the [C_2_O_5_]^2–^ pyrocarbonate anion can exist in different geometries: nondistorted flat [C_2_O_5_]^2–^ is observed in Al_2_(C_2_O_5_) (CO_3_)_2_;^15^ in SrC_2_O_5_ and PbC_2_O_5_ [C_2_O_5_]^2–^, groups are bent at the bridging oxygen atoms;^12,13^ in Na_2_C_2_O_5_, two CO_3_ groups are rotated relative to each other;^17^ Li_2_C_2_O_5_ combines both bending at the bridging oxygen atom and relative rotation of CO_3_ units;^16^ and finally, BaC_2_O_5_ features twisted C_2_O_5_ unit with tetrahedral angle ∠C–O–C = 104°.^14^ From that perspective, the connection of the CO_3_ groups in [C_3_O_7_]^2–^ is similar to their connection in Na_2_C_2_O_5_.

Such [C_3_O_7_]^2–^ anions have never been observed or predicted hitherto. The here-observed [C_3_O_7_]^2–^ anion is the next member of the homologous series of sp^2^-carbonates: carbonate [CO_3_]^2–^–pyrocarbonate [C_2_O_5_]^2–^–tricarbonate [C_3_O_7_]^2–^. Notably, the degree of condensation increases with pressure as the [CO_3_]^2–^ anion is well-known at ambient conditions, pyrocarbonates were synthesized at relatively low pressures of 20–46 GPa,^12–17^ and here-discovered [C_3_O_7_]^2–^ anion is stabilized at 55 GPa. The open question is whether the stabilization of the next members of the sp^2^-carbonate series [C_n_O_2n+1_]^2–^ can be achieved at higher pressures, or tetrafold coordination of carbon would become preferable and mixed sp^2^/sp^3^-carbonates or sp^3^-carbonates would form.

The pyrocarbonate anion has isoelectronic analogs formed by carbon’s neighbors, boron and nitrogen, [B_2_O_5_]^4–^ anion and neutral N_2_O_5_. Isoelectronic analogs to tricarbonate [C_3_O_7_]^2–^ would be the [B_3_O_7_]^5–^ anion and the [N_3_O_7_]^+^ cation. The [N_3_O_7_]^+^ cation has never been observed so far. Borates containing the [B_3_O_7_]^5–^ anion are known, and this anion exists in two distinct geometries. The first type of [B_3_O_7_]^5–^anion represents a ring composed of two trigonal BO_3_ groups and one tetrahedral BO_4_ unit (Figure S2a). In contrast, the second type is geometrically similar to the [C_3_O_7_]^2–^ anion, consisting of three trigonal BO_3_ groups connected by bridging oxygen atoms, although the orientation of the BO_3_ units differs (Figure S2b,c). It is worth noting that the first type is more common in borate crystal chemistry,^19,20^ whereas the second type has so far been identified in only one compound, PbCu_6_B_6_O_16_.^21^ This suggests that mixed sp^2^/sp^3^-[C_3_O_7_]^2–^ anions may also be discovered in the future.

For cross-validation of the K_2_C_3_O_7_ crystal structure and to get a deeper insight into the stability and physical properties of the novel compound, we performed density functional theory (DFT) calculations (see Methods in Supporting Information). We carried out variable cell structural relaxation for K_2_C_3_O_7_ at 55 GPa and found that the relaxed unit cell volume and unit cell parameters closely reproduce the corresponding experimental values (Table S4). The calculated bond lengths and bond orders (crystal orbital bond index, ICOBI)^22^ are also in good agreement with the experimental data (Table S3). The phonon dispersion relations calculated in the harmonic approximation show that K_2_C_3_O_7_ is dynamically stable at a synthesis pressure of 55 GPa (Figure S3a). DFT calculations indicate that K_2_C_3_O_7_ exhibits a semiconducting behavior (Figure S4). We also calculated a Raman spectrum of K_2_C_3_O_7_ to provide information on signature vibrations of the [C_3_O_7_]^2–^ anion (Supporting Information discussion 2).

To trace the structure behavior at different pressures, the full variable-cell structure relaxations for K_2_C_3_O_7_ were performed with 10 GPa pressure steps between 0 and 120 GPa. The structure can be relaxed at each pressure below 80 GPa down to 1 bar without significant structural changes, although it becomes dynamically unstable at 1 bar (Figure S3b). Interestingly, DFT calculations suggest a phase transition between 80 and 90 GPa, since the initial structure becomes unstable at 90 GPa and relaxes toward another nearby local minimum (Table S5). The relaxed structure obtained at 90 GPa exhibits pronounced structural rearrangements caused by the displacement of C1 and C3 carbon atoms, altering their coordination polyhedra from planar trigonal to tetrahedral (Figure 3a,b). Notably, at 80 GPa, there is no covalent interaction between C1–O7 or C3–O1, as indicated by the relatively long distances of *d*_1_ = 1.949 and *d*_2_ = 1.997 Å, respectively. However, at 90 GPa, a significant drop in these distances to *d*_1_ = 1.442 and *d*_2_ = 1.432 Å clearly indicates the formation of covalent bonds between C1–O7 and C3–O1 (Figure 3c). It is also accompanied by a notable change in the unit cell shape (Figures S5 and S6), with an especially drastic drop in the *a* lattice parameter—the primary direction of carbon atom displacement.

**Figure 3:**
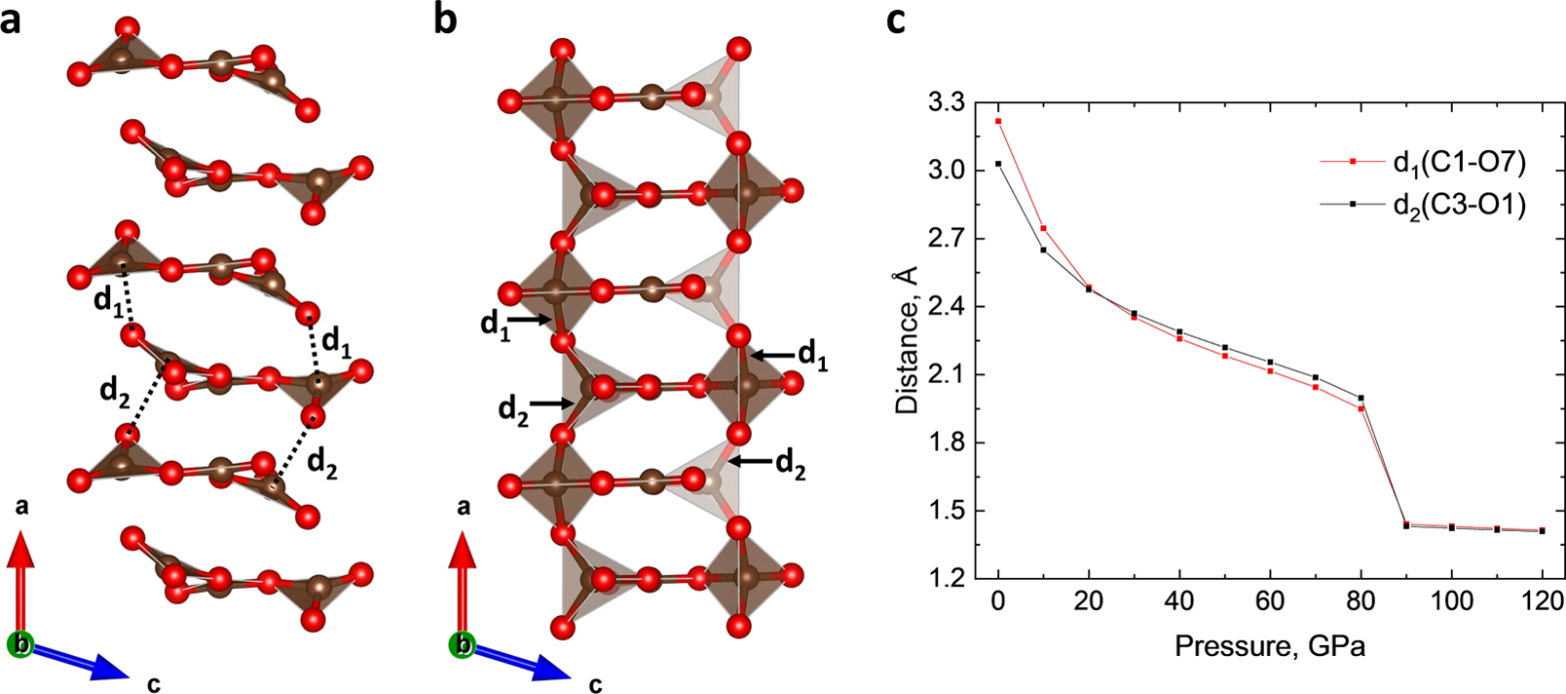
(a) Stacks of isolated [C_3_O_7_]^2–^ anions along the *a* direction in the DFT-relaxed structure of K_2_C_3_O_7_ at 80 GPa. (b) Structural transformation of the stack viewed along the *a* direction in the DFT-relaxed structure of K_2_C_3_O_7_ at 90 GPa. (c) Pressure dependence of *d*_1_(C1–O7) and *d*_2_(C3–O1) distances.

The volume-pressure dependence of DFT-relaxed structures of K_2_C_3_O_7_ in the pressure range of 0–80 GPa was fitted with a third-order Birch–Murnaghan equation of state (Figure 4a), leading to the bulk modulus of *K*_0_(K_2_C_3_O_7_) = 24.5(5) GPa with *Ḱ* = 5.27(8). The obtained bulk modulus is lower than typical values of bulk moduli for pyrocarbonates (*K*_0_ = 32–41 GPa), which in turn are lower than typical values for carbonates featuring the [CO_3_]^2–^ anion. This observation demonstrates that an increase in the degree of condensation in [CO_3_]^2–^—[C_2_O_5_]^2–^—[C_3_O_7_]^2–^ anionic series leads to a bulkier anion, less dense packing, and higher compressibility.

**Figure 4:**
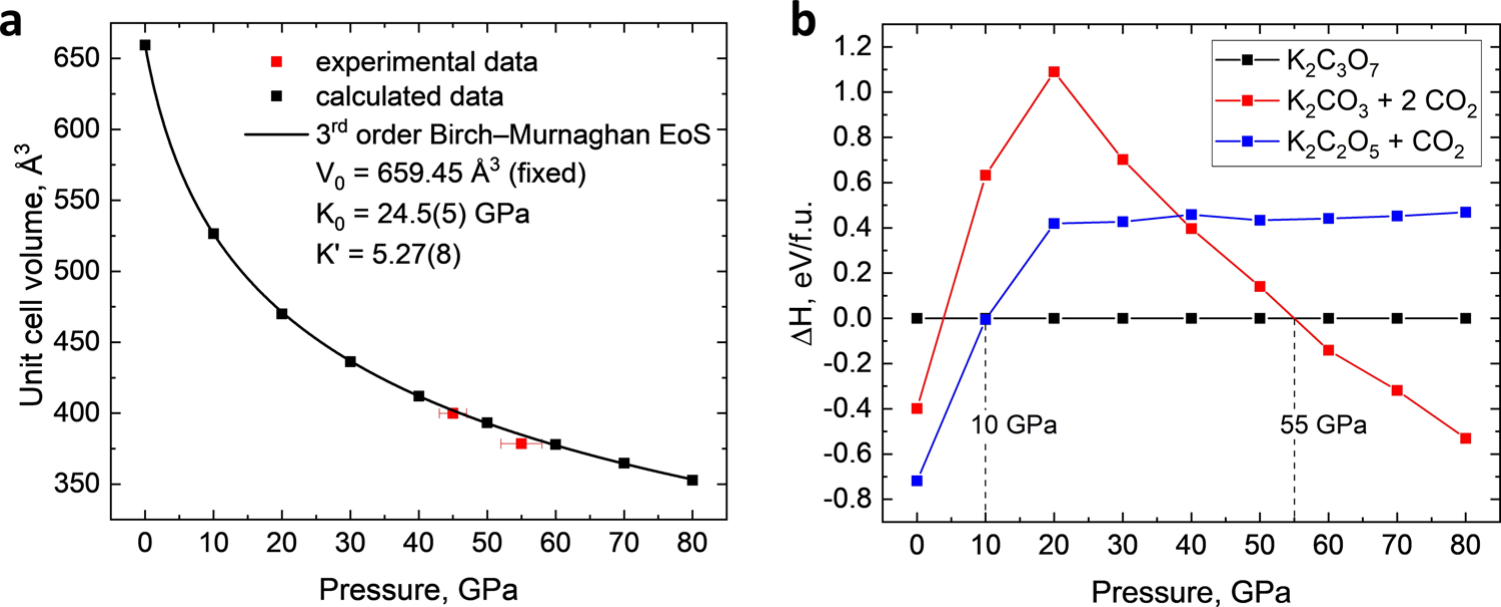
(a) Experimental (red point) and calculated (black points) pressure dependence of the K_2_C_3_O_7_ unit cell volume. The black curve is the fit of the calculated P–V data using a 3rd order Birch–Murnaghan equation of state, yielding *K*_0_ = 24.5(5) GPa, *Ḱ* = 5.27(8). (b) Static enthalpies of K_2_CO_3_ + 2 CO_2_ and K_2_C_2_O_5_ + CO_2_ mixtures relative to the static enthalpy of K_2_C_3_O_7_. Notes: at each pressure point, the thermodynamically stable polymorph of each component (K_2_CO_3_,^23^ K_2_C_2_O_5_,^24^ and CO_2_^25^), as listed in Table S6, was used in the calculations. The calculated enthalpy difference between the K_2_CO_3_ + 2 CO_2_ and K_2_C_2_O_5_ + CO_2_ mixtures is consistent with the results of Banaev et al.,^24^ where possible temperature effects and the impact of CO_2_ entropy on phase boundaries are discussed.

To estimate the pressure range in which the formation of K_2_C_3_O_7_ becomes thermodynamically favorable, we calculated the static enthalpies of K_2_C_3_O_7_ and its competing mixtures—K_2_CO_3_ + 2 CO_2_ and K_2_C_2_O_5_ + CO_2_—across a pressure range of 0–80 GPa (Figure 4b). Various polymorphs of K_2_CO_3_,^23^ K_2_C_2_O_5_,^24^ and CO_2_,^25^ stable at specific pressures, were used in the calculations (Table S6). The results indicate that K_2_C_3_O_7_ is thermodynamically favorable between 10 and 55 GPa at 0 K. Below 10 GPa, the combination of *C*2-K_2_C_2_O_5_ ^24^ and CO_2_–I is more stable, whereas above 55 GPa, *C*2/*c*-K_2_CO_3_ and CO_2_–V become favorable. These findings suggest the possibility of target synthesis of K_2_C_3_O_7_ via direct K_2_CO_3_+CO_2_ reaction at pressures between 10 and 55 GPa. Our experiment at 45(2) GPa provides strong support for this conclusion, although it does not constitute a systematic validation of the proposed pressure range.

## Conclusions

In this study, K_2_C_3_O_7_—the first inorganic tricarbonate salt—was discovered in laser-heated diamond anvil cells. It was synthesized at temperatures up to 3000 K either by oxidation of diamond at 55(3) GPa or targetedly from K_2_CO_3_ and CO_2_ at 45(2) GPa. The observation of K_2_C_3_O_7_ under different chemical environments and pressures indicates that it is thermodynamically favorable over a broad range of chemical potentials and P–T conditions.

The crystal structure of K_2_C_3_O_7_ features nonplanar [C_3_O_7_]^2–^ anions built of three condensed planar CO_3_ units rotated with respect to each other. This anion extends the homologous series of sp^2^-carbonates: [CO_3_]^2–^–[C_2_O_5_]^2–^–[C_3_O_7_]^2–^. Raman spectroscopy establishes the characteristic vibrational fingerprint of the [C_3_O_7_]^2–^ anion, providing a reference for the identification of tricarbonates in future studies.

DFT calculations corroborate the experimental observations, indicating that K_2_C_3_O_7_ is a semiconductor and a relatively soft material, more compressible than typical carbonates and pyrocarbonates. They also suggest that K_2_C_3_O_7_ is thermodynamically stable over a wide pressure range of 10–55 GPa. Between 80 and 90 GPa, a phase transition is predicted, associated with polymerization of the [C_3_O_7_]^2–^ units, accompanied by trigonal-to-tetrahedral coordination changes at two carbon atoms.

These findings highlight the rich structural chemistry accessible to carbonates under compression and suggest that additional sp^2^- and mixed sp^2^/sp^3^-carbonates may be stabilized at high pressure.

## Supplementary Material



## Data Availability

CCDC 2495053 and 2530561 contain the supplementary crystallographic data for this paper. These data can be obtained from CCDC’s and FIZ Karlsruhe’s free service for viewing and retrieving structures (https://www.ccdc.cam.ac.uk/structures/).

## Notes

Methods description, details on the serendipitous synthesis of K_2_C_3_O_7_, Raman characterization of K_2_C_3_O_7_, details of structure refinement, bond order analysis, DFT-relaxed structure parameters, list of polymorphs used in enthalpy calculations, resonance forms of the [C_3_O_7_]^2–^ anion, comparison of the [C_3_O_7_]^2–^ anion with [B_3_O_7_]^5–^ anions, phonon dispersion curves, electron density of states, dependence of the unit cell parameters and volume on the pressure for DFT-relaxed structure, and additional references.^26–55^ (PDF)
