# Supplementary material for: Inorganic Tricarbonate: High-Pressure Synthesis and Structure of K2C3O7
Source: J Am Chem Soc. 2026 Mar 24;148(15):15586–92. doi: 10.1021/jacs.5c19614 (PMC13107454; doi:10.1021/jacs.5c19614)
Supplement: Supplementary file 1 [file ja5c19614_si_001.pdf]

Supporting information

Inorganic Tricarbonate: High-Pressure Synthesis and Structure of $K_2C_3O_7$

Andrey Aslandukov[1*], Alena Aslandukova[1], Fariia I. Akbar[1], Yuqing Yin[2], Ridvan Akilli[1], Gaston Garbarino[3], Dominik Spahr[4], Bjoern Winkler[4], Maxim Bykov[1], Natalia Dubrovinskaia[2], Leonid Dubrovinsky[5*]

[1] Institute of Inorganic and Analytical Chemistry, Goethe University Frankfurt, 60438 Frankfurt, Germany

[2] Material Physics and Technology at Extreme Conditions, Laboratory of Crystallography, University of Bayreuth, 95440 Bayreuth, Germany

[3] European Synchrotron Radiation Facility, 38000 Grenoble, France

[4] Institute of Geosciences, Goethe University Frankfurt, 60438 Frankfurt, Germany

[5] Bavarian Research Institute of Experimental Geochemistry and Geophysics (BGI), University of Bayreuth, 95440 Bayreuth, Germany

Table of Contents

Methods 2
Supplementary discussion 1. Details on the serendipitous synthesis of $K_2C_3O_7$ 5
Supplementary discussion 2. Raman characterization of $K_2C_3O_7$ 6
Supplementary Tables 8
Supplementary Figures 12
References 15

Methods

Sample preparation. For the first experiment, a BX90-type large X-ray aperture DAC[1] equipped with Boehler-Almax type diamonds[2] (culet diameter of 250 μm) was used in the experiment. The sample chamber was formed by pre-indenting a rhenium gasket to 25 μm thickness and laser-drilling a hole of 120 μm in diameter in the center of the indentation. The sample chamber was loaded with $KClO_3$ (99.9%, Sigma Aldrich) and a small piece of boron-doped diamond (BDD, doping level 2000 ppm, GH Diamond Tools Co. Ltd.). No pressure-transmitting medium was used. The sample was compressed first to 22(2) GPa and laser-heated up to 3000(500) K and then compressed to 55(3) GPa and re-heated up to 3000(500) K. The heating was achieved using a custom-built double-sided laser-heating system at the BGI equipped with two Nd:YAG lasers (λ = 1064 nm) and an IsoPlane SCT 320 spectrometer with a 1024×2560 PI-MAX 4 camera for the collection of thermal emission spectra from the heated spot.[3] The temperature during the laser heating was determined by fitting the sample's thermal emission spectra to the grey body approximation of Planck's radiation function in a wavelength range from 570 to 830 nm.

The second experiment was carried out in a Boehler-Almax design DAC[2] equipped with Boehler-Almax type diamonds[2] (culet diameter of 250 μm). The sample chamber was formed by pre-indenting a rhenium gasket to 36 μm thickness and laser-drilling a hole of 90 μm in diameter in the center of the indentation. Powder of K_2CO_3 (99.995%, Sigma Aldrich) was pre-dried in the glovebox at 220°C for 40 minutes, then a selected crystal of K_2CO_3 with dimensions of ~15×15×15 μm^3 was placed in the gasket hole in the glovebox. The DAC was sealed in a glovebox and subsequently transferred to a custom-built cryogenic loading system[4], where CO_2 gas was cryogenically loaded into the cell. Then the sample was compressed to 53(2) GPa and laser-heated from both sides using a custom-built set-up equipped with a Coherent Diamond K-250 pulsed CO_2 laser (λ = 10600 nm).[5] The maximum temperature achieved during the laser-

heating was 3000(500) K, which was determined by the two-color pyrometer method, employing Planck and Wien fits.[6] The duration of heating was 30 minutes. After laser-heating pressure dropped to 45(2) GPa.

The pressure in the DACs was determined using the Raman signal from the diamond anvils[7] and monitored additionally by X-ray diffraction of the Re gasket edge using the rhenium equation of state.[8]

Raman spectroscopy. The Raman spectrum was collected using a dedicated setup comprised of WITec UHTS300 spectrometer (spectral resolution 0.1 cm^{-1}) with a motorized stage alpha300R confocal microscope, a DR316B low dark-current deep depletion CCD camera, a 532-nm excitation UHTS300S_GREEN_NIR laser source and 100mW of its power with a 50x/0.35 Olympus SLMPL objective.

X-ray diffraction. The X-ray diffraction studies were done at the ID15b beamline (λ = 0.4099 Å) and ID11 beamline (λ = 0.2844 Å) of the Extreme Brilliant Source European Synchrotron Radiation Facility (EBS-ESRF). At ID15b, the X-ray beam was focused down to 2 × 1 μm^2 and data were collected with an Eiger2X CdTe 9M detector. At ID11, the X-ray beam was focused down to 1 × 0.8 μm^2 and data were collected with an Eiger2X CdTe 4M detector. In order to determine the position of the polycrystalline sample on which the single-crystal X-ray diffraction acquisition is obtained, a full X-ray diffraction mapping of the pressure chamber was carried out. The sample position displaying the most and the strongest single-crystal reflections belonging to the phase of interest was chosen for the collection of single-crystal data, collected in step-scans of 0.5° from −36° to +36°. The CrysAlis^{Pro} software package[9] was used for the analysis of the single-crystal XRD data (peak hunting, indexing, data integration, frame scaling, and absorption correction). To calibrate an instrumental model in the CrysAlis^{Pro} software, i.e., the sample-to-detector distance, detector's origin, offsets of the goniometer angles, and rotation of both the X-ray beam and detector around the instrument axis, we used a single crystal of orthoenstatite [$(Mg_{1.93}Fe_{0.06})(Si_{1.93},Al_{0.06})O_6$, *Pbca* space group, a = 8.8117(2) Å, b = 5.18320(10) Å, and c = 18.2391(3) Å]. The DAFi program was used for the search of reflection groups belonging to individual single crystal domains.[10] Using the OLEX2 software package,[11] the structures were solved with the ShelXT structure solution program[12] using intrinsic phasing and refined with the ShelXL[13] refinement package using least-squares minimization. Crystal structure visualization was made with the VESTA software.[14]

Theoretical Calculations. First-principles calculations were performed using the framework of density functional theory (DFT) as implemented in the Vienna Ab initio Simulation Package (VASP).[15] To expand the electronic wave function in plane waves, we used the Projector-Augmented-Wave (PAW) method.[16] The Generalized Gradient Approximation (GGA) functional is used for calculating the exchange-correlation energies, as proposed by Perdew–Burke–Ernzerhof (PBE).[17] The PAW potentials with the following valence configurations of 3*s*3*p*4*s* for K (“K_sv”), and 2*s*2*p* for C (“C”) and O (“O”) were used In geometry optimization, we used a Gamma centered 12×6×8 k-mesh, and the plane-wave kinetic energy cutoff was set to 750 eV, with which total energies are converged to better than 2 meV/atom. The electronic convergence criterion was set to $\Delta E = 10^{-8}$ eV. For Brillouin zone integrations, the tetrahedron smearing method with Blöchl corrections (ISMEAR = -5) was used.[18] The phonon frequencies and phonon band structure calculations were performed in the harmonic approximation using the finite displacement method, as implemented in PHONOPY software[19] for 2×1×2 supercell with respectively adjusted *k*-points: 6×6×6. Chemical bonding analyses were conducted using the crystal orbital bond index (COBI) method[20] implemented in the LOBSTER package. [21] Equation of state and static enthalpy calculations were performed via variable-cell structural relaxations between 1 bar and 150 GPa. The theoretical equation of state of $K_2C_3O_7$ was obtained by fitting the pressure−volume data using the EoSFit7-GUI.[22] In our static calculations, the effect of zero-point motion and temperature was neglected.

In order to compute Raman spectra, calculations were carried out within the framework of density functional theory (DFT), employing the Perdew-Burke-Ernzerhof (PBE) exchange-correlation functional and the plane wave/pseudopotential approach implemented in the CASTEP simulation package.[17,23,24] “On the fly” norm-conserving pseudopotentials generated using the descriptors in the CASTEP database were employed in conjunction with plane waves up to a kinetic energy cutoff of 1020~eV. The accuracy of the pseudopotentials is well established.[25] A Monkhorst-Pack grid was used for Brillouin zone integrations.[26] We used a distance between grid points of < 0.023 Å^{-1}. Convergence criteria for geometry optimization included an energy change of < 5×10^{-6} eV/atom between steps, a maximal force of < 0.008 eV/Å and a maximal component of the stress tensor < 0.02 GPa. Phonon frequencies were obtained from density functional perturbation theory (DFPT) calculations.[27,28] Raman intensities were computed using DFPT with the “2n+1” theorem approach.[29]

Supplementary discussion 1. Details on the serendipitous synthesis of $K_2C_3O_7$

As described in the “Methods/Sample preparation” section, in the first experiment, a diamond anvil cell loaded with $KClO_3$ and boron-doped diamond (BDD) was used. First, the sample chamber was compressed to 22(2) GPa and the sample was laser heated (**Fid. SD1a,b**). X-ray diffraction data revealed the formation of an orthorhombic phase with a = 5.915(14) Å, b = 6.413(5) Å, and c = 6.691(5) Å lattice parameters. Only lattice parameters could be extracted, since this phase was found to be X-ray-sensitive and the compound decomposed during SCXRD data collection. Most likely, it is a high-pressure polymorph of $KClO_3$ that decomposes into KCl and O_2 under X-rays. Such behavior of $KClO_3$ was previously reported.[30] The formation of O_2 after X-ray exposure can also be seen optically, since the sample got a light-orange colour in the square region where the XRD map was collected, and dark-orange in places where SCXRD datasets were collected (**Fid. SD1c**).

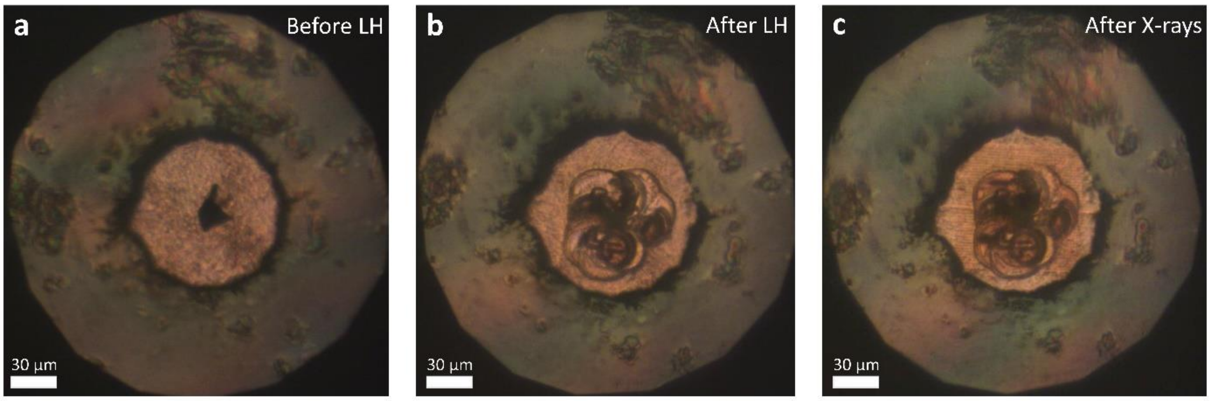

Fig. SD1. Microphotographs of the sample chamber containing a black piece of BDD embedded in transparent $KClO_3$ at 22(2) GPa (a) before laser-heating, (b) after laser-heating, and (c) after X-ray exposure.

Then the sample, consisting of $KClO_3$, KCl, O_2 and BDD, was compressed to 55(3) GPa and re-heated. Single-crystal X-ray diffraction reveals the well-crystalline grains of at least four phases. The first phase is a B2-KCl (a = 3.1119(14) Å, V = 30.14(2) Å^3). The second phase is P-3c1-KCl_3 (a = 6.7537(15) Å, c = 8.3659(17) Å, V = 330.47(16) Å^3), which is known under high pressure.[31] The third phase has a tetragonal lattice (a = 5.4771(15) Å, c = 3.390(3) Å, V = 101.70(9) Å^3) and was solved as $KClO_4$ in $P4/n$ spacegroup: this high-pressure $KClO_4$ polymorph is hitherto unknown and will be published elsewhere. The fourth phase, $K_2C_3O_7$, is described in the present manuscript in detail.

The only sources of carbon in the system are BDD and diamond anvils, so the formation of potassium tricarbonate is a result of a redox reaction. Taking into account the presence of

strong oxidizers in the system and formation of KCl_3, one can suggest one of the following synthetic routes:

(1) $7\ KClO_3 + 2\ KCl + 9\ C = 3\ K_2C_3O_7 + 3\ KCl_3$

(2) $7\ KClO_4 + 5\ KCl + 12\ C = 4\ K_2C_3O_7 + 4\ KCl_3$

(3) $6\ KCl + 7\ O_2 + 6\ C = 2\ K_2C_3O_7 + 2\ KCl_3$

Supplementary discussion 2. Raman characterization of $K_2C_3O_7$

Raman spectroscopy was used for additional characterization of the $K_2C_3O_7$. In the first experiment at 55(3) GPa, $K_2C_3O_7$ was synthesized as a minor byproduct and its Raman signal could not be unambiguously resolved due to the presence of highly intense signals from other Raman-active phases (KCl_3, $KClO_3$, $KClO_4$, and diamond). However, direct synthesis of $K_2C_3O_7$ from K_2CO_3 and CO_2 at 45(2) GPa produced a sample with a pronounced Raman signal, which also agrees well with the calculated Raman spectrum (**Fig. SD2.1**).

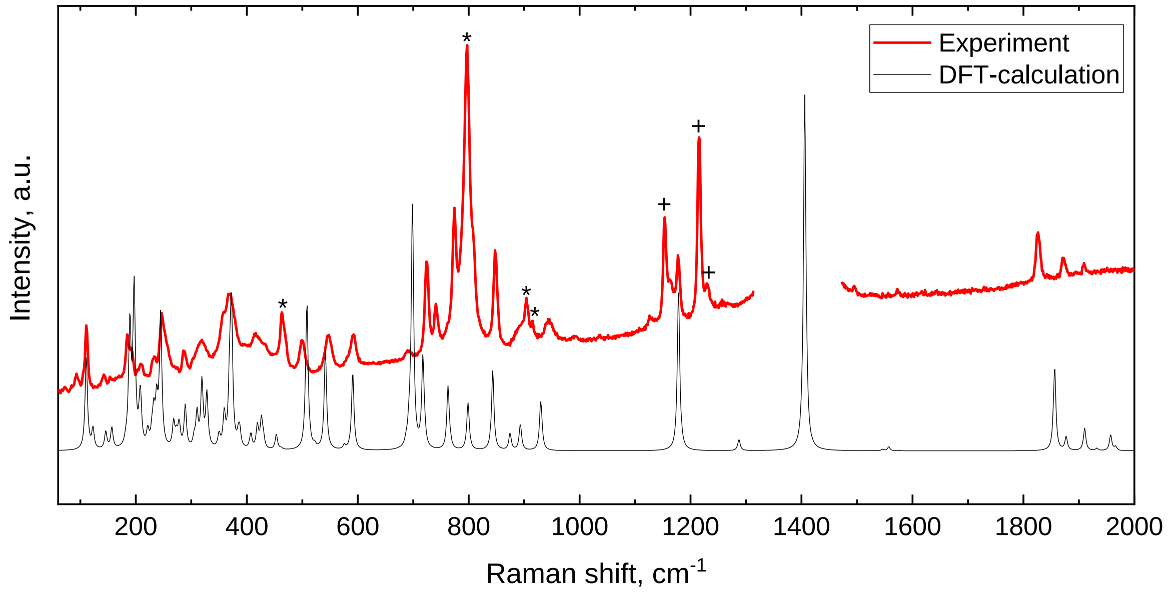

Fig. SD2.1. (a) Experimental spectrum of $K_2C_3O_7$ at 45(2) GPa. Raman modes of CO_2-V are marked by an asterisk (*), and of residual K_2CO_3 by a cross (+). (b) DFT-calculated Raman spectrum of $K_2C_3O_7$ at 45 GPa. The shifts of the calculated Raman spectrum were rescaled by 3.3%.

Since this is the first experimental observation of the $[C_3O_7]^{2-}$ anion, it is important to provide information on its characteristic (fingerprint) vibrational modes to facilitate the identification of other tricarbonates in the future.

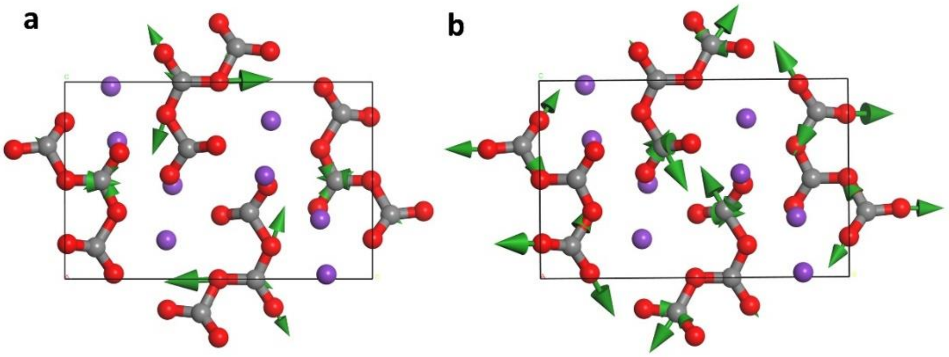

Fig. SD2.2. Eigenvectors of the atomic displacement associated with the signature stretching vibrations of $[C_3O_7]^{2-}$ anion, corresponding to Raman shifts at (a) 1177 cm^{-1} (stretching vibration of central CO_3 group) and (b) 1404 cm^{-1} at 45 GPa (stretching vibration of terminal CO_3 groups).

Two intense Raman bands at 1177 cm^{-1} and 1404 cm^{-1} in the calculated spectrum at 45 GPa correspond to complementary stretching vibrations of the $[C_3O_7]^{2-}$ anion (**Fig. SD2.2**). However, these modes cannot serve as unambiguous fingerprints of the $[C_3O_7]^{2-}$ species in practical experiments. The first band lies within the typical stretching region of the conventional $[CO_3]^{2-}$ anion, while the second overlaps with the diamond Raman peak (**Fig. SD2.1**). Therefore, the most distinctive fingerprint of the $[C_3O_7]^{2-}$ anion is provided by three Raman modes observed in the high-frequency region of 1820–1920 cm^{-1} (**Fig. SD2.1**). The most intense band corresponds predominantly to stretching of the C2-O4 bond of the central CO_3 group (**Fig. SD2.3a**), while the other two correspond to vibrations of terminal CO_3 groups (**Fig. SD2.3b,c**).

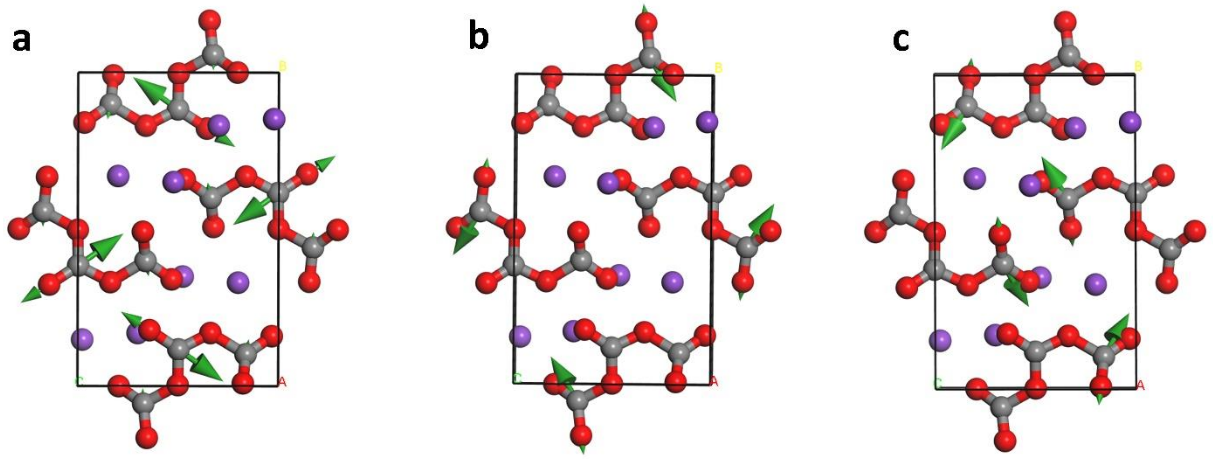

Fig. SD2.3. Eigenvectors of the atomic displacement associated with the signature high-frequency vibrations of $[C_3O_7]^{2-}$ anion, corresponding to observed Raman modes at (a) 1825 cm^{-1}, (b) 1872 cm^{-1} and (c) 1909 cm^{-1} at 45 GPa.

Supplementary Tables

Table S1. Structure refinement details of $K_2C_3O_7$ at 55(3) GPa. The full crystallographic data were deposited to the ICSD under the deposition number CSD 2495053.

Chemical formula	$K_2C_3O_7$
Temperature (K)	293
Pressure (GPa)	55(3)
**Crystal data**	
Mr	226.23
ρ (g/cm^3)	3.971
Crystal system, space group	monoclinic, $P2_1/c$
a (Å)	4.9424(13)
b (Å)	10.946(2)
c (Å)	7.243(2)
β (°)	105.02(3)
V (Å^3)	378.44(18)
Z	4
Radiation type	X-ray, λ = 0.4099 Å
μ (mm^{-1})	0.544
**Data collection**	
No. of measured, independent and observed [I > 2σ(I)] reflections	836/ 659/ 482
R_{int}	2.17%
(sin θ/λ)$_{max}$ (Å^{-1})	0.861
**Refinement**	
R[F^2 > 4σ(F^2)], wR(F^2), GOF	5.89%, 14.53%, 1.034
data/parameters ratio	659/59
$\Delta\rho_{max}$, $\Delta\rho_{min}$ (e Å^{-3})	0.716, -0.807

Atomic positions and equivalent isotropic (or isotropic) ADPs

Atom	Wyckoff site	Fractional atomic coordinates			U_{iso} or U_{eq} (Å^2)
		x	y	z	
K1	4*e*	0.0265(3)	0.34981(10)	0.47541(19)	U_{eq} = 0.0118(3)
K2	4*e*	0.6080(3)	0.66947(9)	0.80299(19)	U_{eq} = 0.0117(3)
O1	4*e*	0.1977(9)	0.5085(3)	0.3220(6)	U_{iso} = 0.0132(8)
O2	4*e*	0.4669(9)	0.6601(3)	0.4723(6)	U_{iso} = 0.0126(8)
O3	4*e*	0.2982(9)	0.6700(3)	0.1627(6)	U_{iso} = 0.0123(7)
O4	4*e*	0.1086(9)	0.6771(3)	-0.1428(6)	U_{iso} = 0.0136(8)
O5	4*e*	0.2499(10)	0.5025(3)	-0.0003(7)	U_{iso} = 0.0176(9)
O6	4*e*	0.1992(9)	0.3338(3)	-0.1555(7)	U_{iso} = 0.0129(7)
O7	4*e*	0.3024(9)	0.5001(3)	-0.2991(7)	U_{iso} = 0.0128(7)
C1	4*e*	0.3355(13)	0.6006(5)	0.3381(9)	U_{iso} = 0.0122(9)
C2	4*e*	0.2078(13)	0.6186(5)	-0.0009(8)	U_{iso} = 0.0126(9)
C3	4*e*	0.2393(12)	0.4425(5)	-0.1729(8)	U_{iso} = 0.0120(10)

Table S2. Structure refinement details of $K_2C_3O_7$ at 45(2) GPa. The full crystallographic data were deposited to the ICSD under the deposition number CSD 2530561.

Chemical formula	$K_2C_3O_7$
Temperature (K)	293
Pressure (GPa)	45(2)
**Crystal data**	
Mr	226.23
ρ (g/cm^3)	3.757
Crystal system, space group	monoclinic, $P2_1/c$
a (Å)	5.0869(17)
b (Å)	11.1050(7)
c (Å)	7.365(4)
β (°)	106.00(5)
V (Å^3)	399.9(3)
Z	4
Radiation type	X-ray, λ = 0.28439 Å
μ (mm^{-1})	0.218
**Data collection**	
No. of measured, independent and observed [I > 2σ(I)] reflections	1351/ 708/ 619
R_{int}	1.58%
(sin θ/λ)$_{max}$ (Å^{-1})	0.957
**Refinement**	
R[F^2 > 4σ(F^2)], wR(F^2), GOF	3.67%, 9.86%, 1.070
data/parameters ratio	708/59
$\Delta\rho_{max}$, $\Delta\rho_{min}$ (e Å^{-3})	0.574, -0.408

Atomic positions and equivalent isotropic (or isotropic) ADPs

Atom	Wyckoff site	Fractional atomic coordinates			U_{iso} or U_{eq} (Å^2)
		x	y	z	
K1	4*e*	0.02792(19)	0.34995(4)	0.47565(18)	U_{eq} = 0.0099(5)
K2	4*e*	0.6038(2)	0.66979(4)	0.80005(18)	U_{eq} = 0.0092(5)
O1	4*e*	0.2010(7)	0.50912(15)	0.3227(6)	U_{iso} = 0.0081(3)
O2	4*e*	0.4654(7)	0.65977(16)	0.4684(7)	U_{iso} = 0.0097(4)
O3	4*e*	0.2901(7)	0.66825(15)	0.1614(7)	U_{iso} = 0.0090(4)
O4	4*e*	0.1053(7)	0.67585(16)	-0.1410(6)	U_{iso} = 0.0094(4)
O5	4*e*	0.2484(6)	0.50223(15)	-0.0007(6)	U_{iso} = 0.0096(4)
O6	4*e*	0.2053(7)	0.33521(15)	-0.1548(6)	U_{iso} = 0.0089(4)
O7	4*e*	0.3004(7)	0.50022(15)	-0.2957(6)	U_{iso} = 0.0087(4)
C1	4*e*	0.3328(9)	0.6010(2)	0.3366(8)	U_{iso} = 0.0084(4)
C2	4*e*	0.2048(9)	0.61818(19)	-0.0014(8)	U_{iso} = 0.0069(4)
C3	4*e*	0.2418(9)	0.4430(2)	-0.1733(8)	U_{iso} = 0.0083(4)

Table S3. Bond order analysis at 55 GPa.

Bond	Bond length		Bond order		
	Exp.	Calc.	Bond length analysis	Analysis based on resonance forms	calc. ICOBI
C1-O1	1.205(7)	1.221	1.5	1.44	1.47
C1-O2	1.210(7)	1.219	1.5	1.44	1.48
C3-O6	1.218(7)	1.228	1.5	1.44	1.45
C3-O7	1.216(8)	1.226	1.5	1.44	1.46
C2-O4	1.202(7)	1.206	1.5	1.50	1.58
C1-O3	1.450(8)	1.448	1	1.125	0.75
C3-O5	1.401(8)	1.411	1	1.125	0.80
C2-O3	1.283(7)	1.296	1.25	1.25	1.11
C2-O5	1.288(7)	1.302	1.25	1.25	1.06

Table S4. Experimentally determined crystallographic data for $K_2C_3O_7$ phase at 55 GPa in comparison with the corresponding DFT-relaxed structure. Note that pressure was fixed in theoretical simulations, while volumes of the unit cells, lattice parameters and equilibrium state parameters were calculated.

	Exp.	Calc.
**Space group**	$P2_1/c$	$P2_1/c$
**Volume**	378.44(18) Å^3	385.22 Å^3
**Lattice parameters**	a = 4.9424(13) Å<br>b = 10.946(2) Å<br>c = 7.243(2) Å<br>β = 105.02(3)°	a = 4.9631 Å<br>b = 11.025 Å<br>c = 7.3297 Å<br>β = 106.16°

Table S5. Crystallographic data for DFT-relaxed $K_2C_3O_7$ structures at 80 and 90 GPa, spanning the pressure range of a phase transition.

**Pressure**		**80 GPa**			**90 GPa**		
**Space group**		$P2_1/c$			$P2_1/c$		
**Volume**		352.75 Å^3			334.11 Å^3		
**Lattice parameters**		a = 4.76154 Å<br>b = 10.768 Å<br>c = 7.1773 Å<br>β = 106.55°			a = 4.5050 Å<br>b = 10.804 Å<br>c = 7.1796 Å<br>β = 107.038°		
**Atom positions**	**Atom**	**Fractional atomic coordinates**			**Fractional atomic coordinates**		
		**x**	**y**	**z**	**x**	**y**	**z**
	**K1**	0.01478	0.35037	0.47639	0.01343	0.34658	0.46988
	**K2**	0.61575	0.67154	0.80868	0.64409	0.67323	0.81018
	**O1**	0.18219	0.50608	0.31603	0.14445	0.50784	0.30070
	**O2**	0.47559	0.65511	0.47966	0.49246	0.64295	0.48022
	**O3**	0.30321	0.66988	0.16618	0.33033	0.66658	0.16606
	**O4**	0.12274	0.68103	0.85333	0.16353	0.68451	0.85495
	**O5**	0.24584	0.50124	0.99694	0.24439	0.50192	0.99010
	**O6**	0.19558	0.33019	0.84255	0.17215	0.33089	0.83850
	**O7**	0.32004	0.49730	0.70196	0.34585	0.49336	0.70028
	**C1**	0.34940	0.59544	0.33659	0.40973	0.58442	0.32636
	**C2**	0.21473	0.62007	0.99736	0.24298	0.61972	0.99611
	**C3**	0.22644	0.44156	0.82234	0.15338	0.44297	0.80307

Table S6. Polymorphs of K_2CO_3[32], $K_2C_2O_5$[33], and CO_2[34] used in enthalpy calculations.

Pressure	competing mixture №1	competing mixture №2
1 bar	γ-K_2CO_3 and CO_2-I	*C*2-$K_2C_2O_5$ and CO_2-I
10 GPa	γ-K_2CO_3 and CO_2-I	$P2_1$-$K_2C_2O_5$ and CO_2-I
20 GPa	$P2_1/m$-K_2CO_3 and CO_2-V	$P2_1$-$K_2C_2O_5$ and CO_2-V
30 GPa	$P2_1/m$-K_2CO_3 and CO_2-V	$P2_1$-$K_2C_2O_5$ and CO_2-V
40 GPa	$P2_1/m$-K_2CO_3 and CO_2-V	$P2_1$-$K_2C_2O_5$ and CO_2-V
50 GPa	$P2_1/m$-K_2CO_3 and CO_2-V	$P2_1$-$K_2C_2O_5$ and CO_2-V
60 GPa	$C2/c$-K_2CO_3 and CO_2-V	$P2_1$-$K_2C_2O_5$ and CO_2-V
70 GPa	$C2/c$-K_2CO_3 and CO_2-V	$P2_1$-$K_2C_2O_5$ and CO_2-V
80 GPa	$C2/c$-K_2CO_3 and CO_2-V	$P2_1$-$K_2C_2O_5$ and CO_2-V

Supplementary Figures

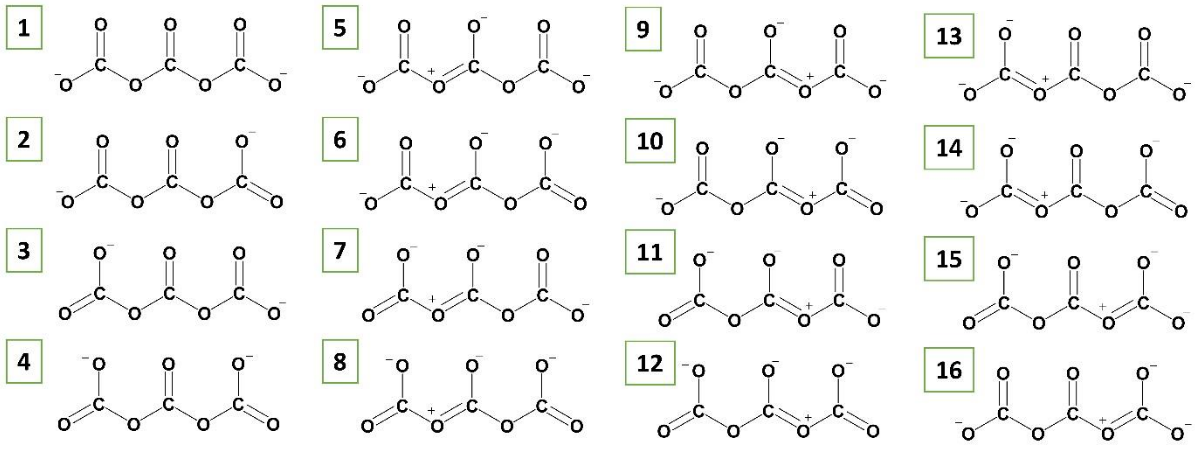

Fig. S1. Resonance forms of $[C_3O_7]^{2-}$ anion. Assuming that all 16 forms are equally probable, the resulting bond order distribution (obtained through averaging of all resonance forms) is already close to the experimentally observed values (Table S2). In the idealized case, this anion adopts a planar geometry with a conjugated π-system. In practice, however, it is nonplanar, most likely due to favorable packing under high pressure. Rotations of the CO_3 groups around the C1-O3 and C3-O5 bonds disrupt complete π-overlap between the orbitals of the C1 and O3 atoms as well as of C3 and O5 atoms, respectively. Consequently, the contributions of resonance forms 13-16 are likely minor under real conditions.

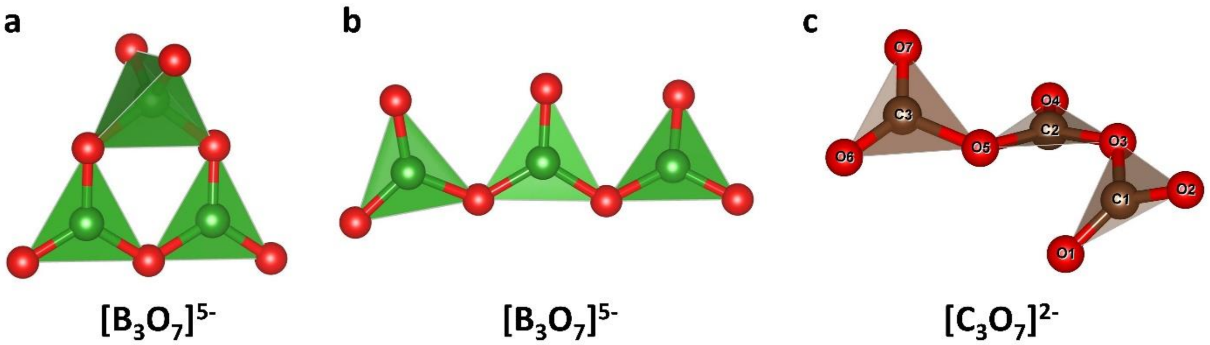

Fig. S2. Geometry of (a) the first type of $[B_3O_7]^{5-}$ anion, (b) the second type of $[B_3O_7]^{5-}$ anion, and (c) the here-discovered $[C_3O_7]^{2-}$ anion.

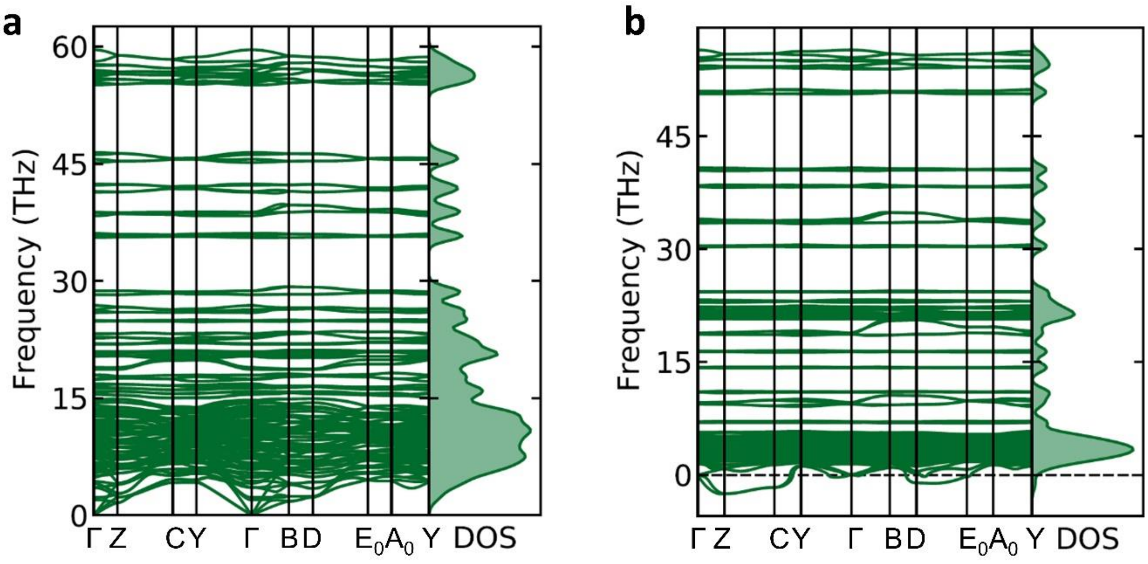

Fig. S3. Phonon dispersion curves for $K_2C_3O_7$ along the high symmetry directions in the Brillouin zone and the phonon density of states at (a) 55 GPa and (b) 1 bar.

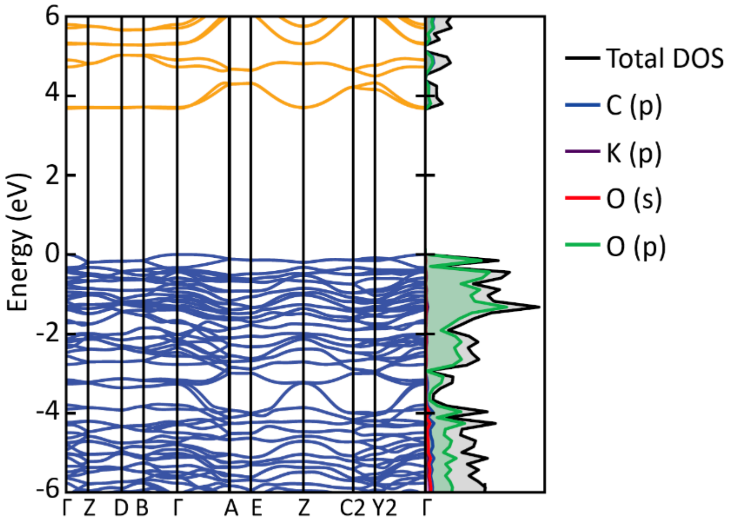

Fig. S4. The electron density of states of $K_2C_3O_7$ at 55 GPa. The energy gap, according to PBE calculations is 3.51 eV.

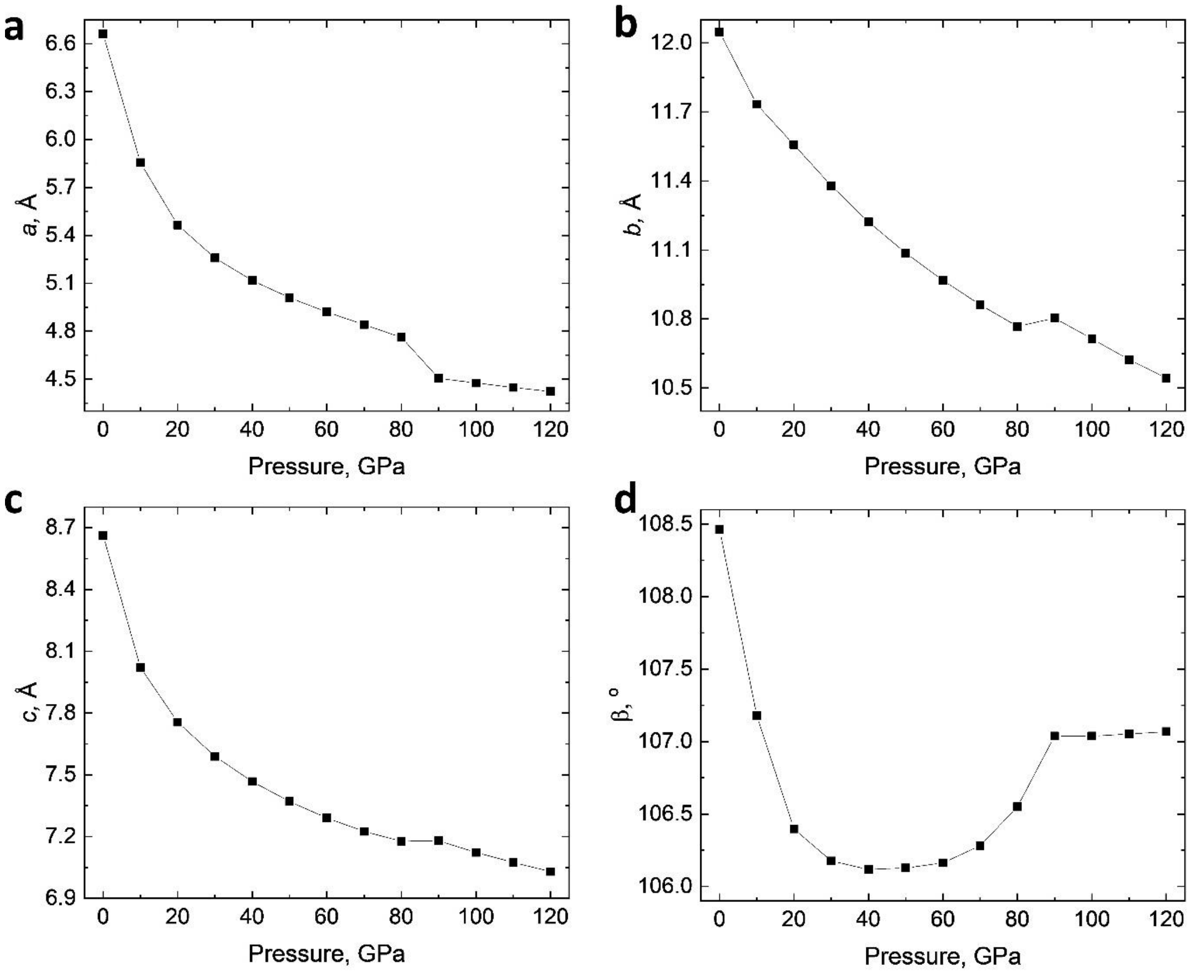

Fig. S5. The dependence of the unit cell parameters on the pressure of $K_2C_3O_7$ obtained from DFT (PBE GGA): (a-c) *a, b, c* lattice parameters and (d) β angle.

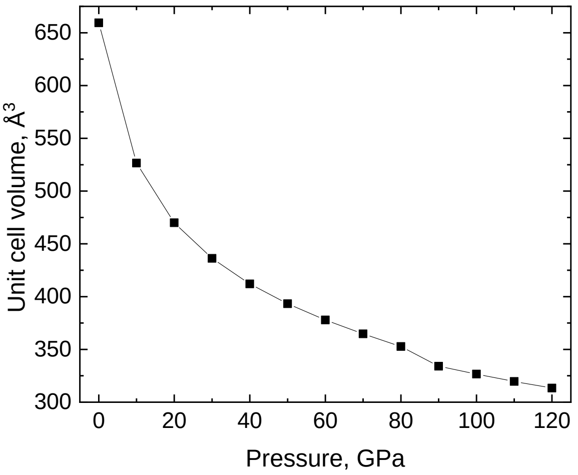

Fig. S6. The pressure-unit cell volume dependence of $K_2C_3O_7$ obtained from DFT (PBE GGA).

References

(1) Kantor, I.; Prakapenka, V.; Kantor, A.; Dera, P.; Kurnosov, A.; Sinogeikin, S.; Dubrovinskaia, N.; Dubrovinsky, L. BX90: A New Diamond Anvil Cell Design for X-Ray Diffraction and Optical Measurements. *Rev. Sci. Instrum.* **2012**, *83* (12), 125102. https://doi.org/10.1063/1.4768541.

(2) Boehler, R. New Diamond Cell for Single-Crystal x-Ray Diffraction. *Rev. Sci. Instrum.* **2006**, *77* (11), 2004–2007. https://doi.org/10.1063/1.2372734.

(3) Fedotenko, T.; Dubrovinsky, L.; Aprilis, G.; Koemets, E.; Snigirev, A.; Snigireva, I.; Barannikov, A.; Ershov, P.; Cova, F.; Hanfland, M.; Dubrovinskaia, N. Laser Heating Setup for Diamond Anvil Cells for in Situ Synchrotron and in House High and Ultra-High Pressure Studies. *Rev. Sci. Instrum.* **2019**, *90* (10), 104501. https://doi.org/10.1063/1.5117786.

(4) Spahr, D.; Bayarjargal, L.; Brüning, L.; Kovalev, V.; Bykova, E.; Bykov, M.; Milman, V.; Mezouar, M.; Winkler, B. Synthesis and Crystal Structure of Anhydrous Di-Iodyl Carbonate $(IO_2)_2[CO_3]$, Hosting I^{5+} Cations. *JACS Au* **2025**, *5*, 4675–4680. https://doi.org/10.1021/jacsau.5c00829.

(5) Bayarjargal, L.; Fruhner, C.-J.; Schrodt, N.; Winkler, B. $CaCO_3$ Phase Diagram Studied with Raman Spectroscopy at Pressures up to 50 GPa and High Temperatures and DFT Modeling. *Phys. Earth Planet. Inter.* **2018**, *281* (March), 31–45. https://doi.org/10.1016/j.pepi.2018.05.002.

(6) Benedetti, L. R.; Loubeyre, P. Temperature Gradients, Wavelength-Dependent Emissivity, and Accuracy of High and Very-High Temperatures Measured in the Laser-Heated Diamond Cell. *High Press. Res.* **2004**, *24* (4), 423–445.

(7) Akahama, Y.; Kawamura, H. Pressure Calibration of Diamond Anvil Raman Gauge to 310 GPa. *J. Appl. Phys.* **2006**, *100* (4), 043516. https://doi.org/10.1063/1.2335683.

(8) Anzellini, S.; Dewaele, A.; Occelli, F.; Loubeyre, P.; Mezouar, M. Equation of State of Rhenium and Application for Ultra High Pressure Calibration. *J. Appl. Phys.* **2014**, *115* (4), 043511. https://doi.org/10.1063/1.4863300.

(9) Rigaku Oxford Diffraction, CrysAlisPro Software System (2015).

(10) Aslandukov, A.; Aslandukov, M.; Dubrovinskaia, N.; Dubrovinsky, L. Domain Auto Finder (DAFi) Program: The Analysis of Single-Crystal X-Ray Diffraction Data from Polycrystalline Samples. *J. Appl. Crystallogr.* **2022**, *55* (5), 1383–1391. https://doi.org/10.1107/S1600576722008081.

(11) Dolomanov, O. V.; Bourhis, L. J.; Gildea, R. J.; Howard, J. A. K.; Puschmann, H. OLEX2: A Complete Structure Solution, Refinement and Analysis Program. *J. Appl. Crystallogr.* **2009**, *42* (2), 339–341. https://doi.org/10.1107/S0021889808042726.

(12) Sheldrick, G. M. SHELXT - Integrated Space-Group and Crystal-Structure Determination. *Acta Crystallogr. Sect. A Found. Crystallogr.* **2015**, *71* (1), 3–8. https://doi.org/10.1107/S2053273314026370.

(13) Sheldrick, G. M. Crystal Structure Refinement with SHELXL. *Acta Crystallogr. Sect. C Struct. Chem.* **2015**, *71* (1), 3–8. https://doi.org/10.1107/S2053229614024218.

(14) Momma, K.; Izumi, F. VESTA 3 for Three-Dimensional Visualization of Crystal, Volumetric and Morphology Data. *J. Appl. Crystallogr.* **2011**, *44* (6), 1272–1276. https://doi.org/10.1107/S0021889811038970.

(15) Kresse, G.; Furthmüller, J. Efficient Iterative Schemes for Ab Initio Total-Energy Calculations Using a Plane-Wave Basis Set. *Phys. Rev. B* **1996**, *54* (16), 11169–11186. https://doi.org/10.1103/PhysRevB.54.11169.

(16) Kresse, G.; Joubert, D. From Ultrasoft Pseudopotentials to the Projector Augmented-Wave Method. *Phys. Rev. B - Condens. Matter Mater. Phys.* **1999**, *59* (3), 1758–1775. https://doi.org/10.1103/PhysRevB.59.1758.

(17) Perdew, J. P.; Burke, K.; Ernzerhof, M. Generalized Gradient Approximation Made Simple. *Phys. Rev. Lett.* **1996**, *77* (18), 3865–3868. https://doi.org/10.1103/PhysRevLett.77.3865.

(18) Blöchl, P. E.; Jepsen, O.; Andersen, O. K. Improved Tetrahedron Method for Brillouin-Zone Integrations. *Phys. Rev. B* **1994**, *49* (23), 16223–16233. https://doi.org/10.1103/PhysRevB.49.16223.

(19) Togo, A.; Oba, F.; Tanaka, I. First-Principles Calculations of the Ferroelastic Transition between Rutile-Type and $CaCl_2$-Type SiO_2 at High Pressures. *Phys. Rev. B - Condens. Matter Mater. Phys.* **2008**, *78* (13), 134106. https://doi.org/10.1103/PhysRevB.78.134106.

(20) Müller, P. C.; Ertural, C.; Hempelmann, J.; Dronskowski, R. Crystal Orbital Bond Index: Covalent Bond Orders in Solids. *J. Phys. Chem. C* **2021**, *125* (14), 7959–7970. https://doi.org/10.1021/acs.jpcc.1c00718.

(21) Nelson, R.; Ertural, C.; George, J.; Deringer, V. L.; Hautier, G.; Dronskowski, R. LOBSTER: Local Orbital Projections, Atomic Charges, and Chemical-Bonding Analysis from Projector-Augmented-Wave-Based Density-Functional Theory. *J. Comput. Chem.* **2020**, *41* (21), 1931–1940. https://doi.org/10.1002/jcc.26353.

(22) Gonzalez-Platas, J.; Alvaro, M.; Nestola, F.; Angel, R. EosFit7-GUI: A New Graphical User Interface for Equation of State Calculations, Analyses and Teaching. *J. Appl. Crystallogr.* **2016**, *49* (4), 1377–1382. https://doi.org/10.1107/S1600576716008050.

(23) Hohenberg, P.; Kohn, W. Inhomogeneous Electron Gas. *Phys. Rev.* **1964**, *136* (3B), B864–B871. https://doi.org/10.1103/PhysRev.136.B864.

(24) Clark, S. J.; Segall, M. D.; Pickard, C. J.; Hasnip, P. J.; Probert, M. I. J.; Refson, K.; Payne, M. C. First Principles Methods Using CASTEP. *Zeitschrift fur Krist.* **2005**, *220* (5–6), 567–570. https://doi.org/10.1524/zkri.220.5.567.65075.

(25) Lejaeghere, K.; Bihlmayer, G.; Björkman, T.; Blaha, P.; Blügel, S.; Blum, V.; Caliste, D.; Castelli, I. E.; Clark, S. J.; Dal Corso, A.; de Gironcoli, S.; Deutsch, T.; Dewhurst, J. K.; Di Marco, I.; Draxl, C.; Dułak, M.; Eriksson, O.; Flores-Livas, J. A.; Garrity, K. F.; Genovese, L.; Giannozzi, P.; Giantomassi, M.; Goedecker, S.; Gonze, X.; Grånäs, O.;

Gross, E. K. U.; Gulans, A.; Gygi, F.; Hamann, D. R.; Hasnip, P. J.; Holzwarth, N. A. W.; Iuşan, D.; Jochym, D. B.; Jollet, F.; Jones, D.; Kresse, G.; Koepernik, K.; Küçükbenli, E.; Kvashnin, Y. O.; Locht, I. L. M.; Lubeck, S.; Marsman, M.; Marzari, N.; Nitzsche, U.; Nordström, L.; Ozaki, T.; Paulatto, L.; Pickard, C. J.; Poelmans, W.; Probert, M. I. J.; Refson, K.; Richter, M.; Rignanese, G.-M.; Saha, S.; Scheffler, M.; Schlipf, M.; Schwarz, K.; Sharma, S.; Tavazza, F.; Thunström, P.; Tkatchenko, A.; Torrent, M.; Vanderbilt, D.; van Setten, M. J.; Van Speybroeck, V.; Wills, J. M.; Yates, J. R.; Zhang, G.-X.; Cottenier, S. Reproducibility in Density Functional Theory Calculations of Solids. *Science.* **2016**, *351* (6280), aad3000. https://doi.org/10.1126/science.aad3000.

(26) Monkhorst, H. J.; Pack, J. D. Special Points for Brillouin-Zone Integrations. *Phys. Rev. B* **1976**, *13* (12), 5188–5192. https://doi.org/10.1103/PhysRevB.13.5188.

(27) Baroni, S.; de Gironcoli, S.; Dal Corso, A.; Giannozzi, P. Phonons and Related Crystal Properties from Density-Functional Perturbation Theory. *Rev. Mod. Phys.* **2001**, *73* (2), 515–562. https://doi.org/10.1103/RevModPhys.73.515.

(28) Refson, K.; Tulip, P. R.; Clark, S. J. Variational Density-Functional Perturbation Theory for Dielectrics and Lattice Dynamics. *Phys. Rev. B - Condens. Matter Mater. Phys.* **2006**, *73* (15), 155114. https://doi.org/10.1103/PhysRevB.73.155114.

(29) Miwa, K. Prediction of Raman Spectra with Ultrasoft Pseudopotentials. *Phys. Rev. B - Condens. Matter Mater. Phys.* **2011**, *84* (9), 094304. https://doi.org/10.1103/PhysRevB.84.094304.

(30) Pravica, M.; Bai, L.; Bhattacharya, N. High-Pressure X-Ray Diffraction Studies of Potassium Chlorate. *J. Appl. Crystallogr.* **2012**, *45* (1), 48–52. https://doi.org/10.1107/S0021889811053957.

(31) Yin, Y.; Aslandukova, A.; Jena, N.; Trybel, F.; Abrikosov, I. A.; Winkler, B.; Khandarkhaeva, S.; Fedotenko, T.; Bykova, E.; Laniel, D.; Bykov, M.; Aslandukov, A.; Akbar, F. I.; Glazyrin, K.; Garbarino, G.; Giacobbe, C.; Bright, E. L.; Jia, Z.; Dubrovinsky, L.; Dubrovinskaia, N. Unraveling the Bonding Complexity of Polyhalogen Anions: High-Pressure Synthesis of Unpredicted Sodium Chlorides Na_2Cl_3 and Na_4Cl_5 and Bromide Na_4Br_5. *JACS Au* **2023**, *3* (6), 1634–1641. https://doi.org/10.1021/jacsau.3c00090.

(32) Gavryushkin, P. N.; Bekhtenova, A.; Lobanov, S. S.; Shatskiy, A.; Likhacheva, A. Y.; Sagatova, D.; Sagatov, N.; Rashchenko, S. V.; Litasov, K. D.; Sharygin, I. S.; Goncharov, A. F.; Prakapenka, V. B.; Higo, Y. High-Pressure Phase Diagrams of Na_2CO_3 and K_2CO_3. *Minerals* **2019**, *9* (10), 599. https://doi.org/10.3390/min9100599.

(33) Banaev, M. V.; Sagatova, D. N.; Sagatov, N. E.; Gavryushkin, P. N. $K_2C_2O_5$ ─The First Finding of Pyrocarbonate Stable at Ambient Pressure. *Cryst. Growth Des.* **2025**, *25* (11), 3786–3792. https://doi.org/10.1021/acs.cgd.5c00200.

(34) Cogollo-Olivo, B. H.; Biswas, S.; Scandolo, S.; Montoya, J. A. Ab Initio Determination of the Phase Diagram of CO_2 at High Pressures and Temperatures. *Phys. Rev. Lett.* **2020**, *124* (9), 095701. https://doi.org/10.1103/PhysRevLett.124.095701.
